# Endoglin as an Adhesion Molecule in Mature and Progenitor Endothelial Cells: A Function Beyond TGF-β

**DOI:** 10.3389/fmed.2019.00010

**Published:** 2019-01-30

**Authors:** Elisa Rossi, Carmelo Bernabeu, David M. Smadja

**Affiliations:** ^1^Université Paris Descartes, Sorbonne Paris Cité, Paris, France; ^2^Inserm UMR-S1140, Paris, France; ^3^Centro de Investigaciones Biológicas, Consejo Superior de Investigaciones Científicas, Centro de Investigación Biomédica en Red de Enfermedades Raras, Madrid, Spain; ^4^Department of Hematology, AP-HP, Hôpital Européen Georges Pompidou, Paris, France; ^5^Laboratory of Biosurgical Research, Carpentier Foundation, Hôpital Européen Georges Pompidou, Paris, France

**Keywords:** endoglin, endothelial progenitors, EPC, ECFCs, HHT1, integrins, TGF-β

## Abstract

Endoglin (ENG) is a transmembrane glycoprotein expressed on endothelial cells that functions as a co-receptor for several ligands of the transforming growth factor beta (TGF-β) family. ENG is also a recognized marker of angiogenesis and mutations in the endoglin gene are responsible for Hereditary Hemorrhagic Telangiectasia (HHT) type 1, a vascular disease characterized by defective angiogenesis, arteriovenous malformations, telangiectasia, and epistaxis. In addition to its involvement in the TGF-β family signaling pathways, several lines of evidence suggest that the extracellular domain of ENG has a role in integrin-mediated cell adhesion via its RGD motif. Indeed, we have described a role for endothelial ENG in leukocyte trafficking and extravasation *via* its binding to leukocyte integrins. We have also found that ENG is involved in vasculogenic properties of endothelial progenitor cells known as endothelial colony forming cells (ECFCs). Moreover, the binding of endothelial ENG to platelet integrins regulate the resistance to shear during platelet-endothelium interactions under inflammatory conditions. Because of the need for more effective treatments in HHT and the involvement of ENG in angiogenesis, current studies are aimed at identifying novel biological functions of ENG which could serve as a therapeutic target. This review focuses on the interaction between ENG and integrins with the aim to better understand the role of this protein in blood vessel formation driven by progenitor and mature endothelial cells.

## Endoglin Structure And Function

Endoglin (ENG; also known as CD105) is a type I transmembrane glycoprotein predominantly expressed in endothelial cells (ECs) (1, 2) and endothelial colony-forming cells (ECFCs) (3). It is known as an essential co-receptor for the transforming growth factor β (TGF-β) family, playing an important role in angiogenesis. At the cell surface, ENG associates with TGF-β type I receptors, including the ECs-specific ALK1 (activin receptor-like kinase 1) and the ubiquitous ALK5, as well as to a TGF-β type II receptor. In this receptor complex, ENG and ALK1 are able to bind to bone morphogenetic protein 9 (BMP9; also known as growth differentiation factor 2 [GDF2]), a member of the TGF-β family, with much higher affinity than to TGF-β1 and mediate its proliferation signal (4–7).

**Figure 1:**
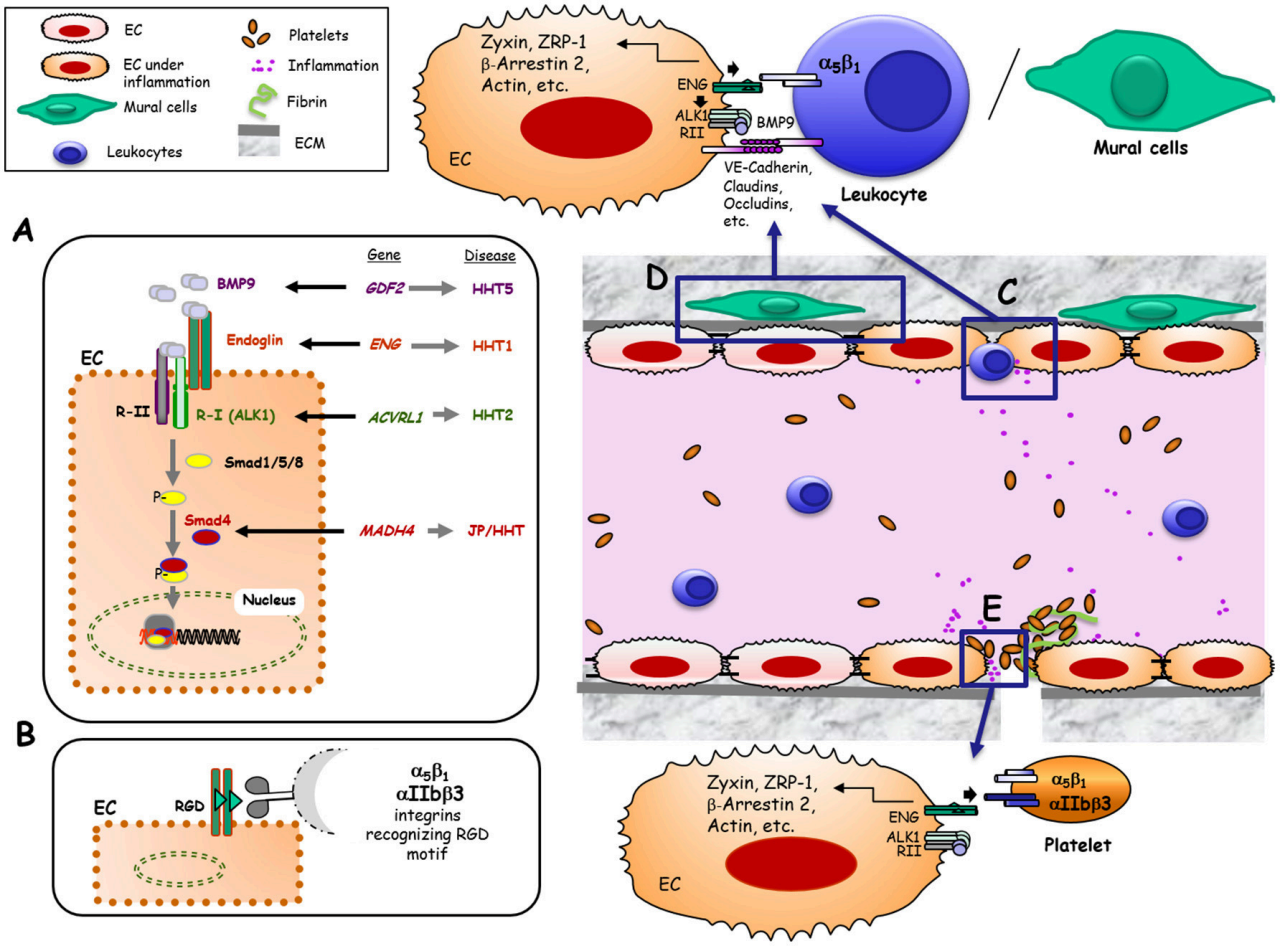
Hypothetical model of Endoglin and its role as TGF-β co-receptor and adhesion molecule in different physiological contexts. **(A)** Canonical pathway that implicates Eng as a co-receptor of TGF-β in ECs. Bone morphogenetic protein 9 (BMP9), and other members of the TGF-β family, bind to an EC receptor complex composed by the type I (R-I) receptor named ALK1 and the type II (R-II) receptor, both exhibiting serine/threonine kinase activity, as well as the auxiliary receptor endoglin. Upon ligand binding, the R-II transphosphorylates ALK1, which subsequently propagates the signal by phosphorylating the receptor-regulated Smad (R-Smad) family of proteins Smad1/5/8. Phosphorylated R-Smads form heteromeric complexes with Smad4, translocating into the nucleus to regulate the transcriptional activity of different target genes. BMP9, Endoglin, ALK1 and Smad4 proteins are encoded by GDF2, ENG, ACVRL1 and MADH4 genes, whose pathogenic mutations give rise to HHT5, HHT1, HHT2, and JPHT, respectively (30). **(B)** Endothelial endoglin as an adhesion molecule involving its RGD sequence when binding to cell surface integrins from leukocytes, VMCs or platelets. **(C)** Leukocyte transmigration through the vessel endothelium. Under inflammatory conditions, different soluble factors are released leading to leukocyte adhesion and transmigration through ECs. This process is mediated, at least, by the interaction between leukocyte integrin α5β1 and endothelial endoglin involving the specific recognition of the RGD motif in Eng (22). **(D)** Adhesion between endothelial cells and vascular mural cells (VMCs). This intercellular association involves, at least, the interaction between integrin α5β1 in VMCs and endothelial endoglin via the specific recognition of the RGD motif in Eng (23). **(E)** Platelet-dependent hemostasis. Endothelial endoglin via its RGD binds to platelet integrins αIIbβ3 and α5β1, contributing to stabilize platelets adhesion to endothelium (24).

Upon ligand binding, the TGF-β signaling receptor complex phosphorylates members of the Smad family of transcription factors which, in turn, undergo nuclear translocation to regulate gene expression (Figure 1A).

In addition to the membrane bound form of endoglin, a circulating form of the ENG ectodomain, was found to be released upon a C-terminal cleavage of ENG by matrix metalloproteinase 14 (MMP-14) (8, 9). Increased levels of circulating endoglin have been detected at early stages of preeclampsia (5, 10) in hypertensive or diabetic patients (11) and in certain cancer patients (12), suggesting its role as a predictive biomarker in these pathologies. Interestingly, a detailed characterization of plasma from women with preeclampsia has revealed that circulating endoglin can be complexed within exosomes, rather than as individual soluble endoglin (13).

A pioneer article on human ENG described that this glycoprotein is present on ECs as a dimer (two subunits of ~90-kDa), each displaying the tripeptide arginine-glycine-aspartic acid (RGD) in the extracellular region of the protein (1). The RGD motif and homologous sequences in mouse and pig are recognition motifs for cell surface integrins present in extracellular matrix proteins such as fibronectin, tenascin, thrombospondin, vitronectin, von Willebrand factor as well as fibrinogen and prothrombin (14–16). Integrin-mediated adhesion is involved in hemostasis, thrombosis, and inflammation, processes in which the endothelium plays a critical role. Therefore, the presence of the RGD motif within a hydrophilic environment (1) and its accessibility in the 3D structure of ENG (7), clearly suggest the involvement of ENG in integrin binding, as postulated in early studies (17, 18). In fact, endothelial endoglin binds to leukocyte integrins, allowing leukocyte extravasation (19) suggesting a possible novel function for ENG independent of TGF-β signaling (Figures 1B,C). Several lines of evidence support the role of ENG in leukocyte trafficking: (i) ENG expression is markedly up regulated in endothelial cells of inflamed tissues with an associated inflammatory cell infiltrate (19); (ii) ENG is up-regulated in the post-ischemic kidney and ENG-haploinsufficient mice are protected from renal ischemia-reperfusion injury, due to a reduction of cellular inflammatory responses (20); (iii) Arterial, venous, and capillary endothelia in lymphoid organs are highly reactive with anti-ENG antibodies and a marked staining pattern is observed in high endothelial venules (21); and (iv) Although ENG is present throughout the vascular endothelium, its expression, as compared to veins or arteries, is stronger in capillaries, where most leukocyte infiltration to organs occurs.

In addition to leukocytes, other cell types present in the circulatory system have also revealed an integrin-mediated adhesion activity toward endothelial endoglin (22–24) (Figures 1D,E). Thus, endoglin plays a role in integrin-mediated adhesion of vascular mural cells to endothelium (23). Also, a new role for endoglin in αIIbβ3 integrin-mediated adhesion of platelets to the endothelium, conferring resistance of adherent platelets to detachment has been described (24) (Figure 1E).

## Vascular Pathophysiology of Endoglin in Hereditary Hemorragic Telangectasia (hht)

Hereditary Hemorrhagic Telangiectasia (HHT) is an autosomal dominant disease with a prevalence of one case per 5,000–8,000 individuals (25, 26). HHT patients develop muco-cutaneous lesions known as telangiectasia in the nose, mouth, and gastrointestinal tract, as well as larger AVMs in major organs such as the lung, liver, and brain (25). The telangiectasia are comprised of fragile vessels that are susceptible to rupture and hemorrhage, which means that HHT patients can suffer recurrent anemia following frequent and severe bleeding episodes. Mutations in the endoglin gene (*ENG*) are responsible for HHT type 1 (HHT1) (27), while mutations in activing receptor-like kinase (*ACVRL1* alias *ALK1*) are responsible for HHT2 (2, 27). Taken together, HHT1 and HHT2 account for more than 80% of all HHT cases, whereas a small number of patients show mutations in SMAD4 (*MADH4*) or in BMP9 gene (*GDF2*), which are responsible for less common HHT variants such as a combined syndrome with juvenile polyposis (JP-HHT) and HHT5, respectively (28, 29). Remarkably, all of these genes code for proteins involved in the TGF-β family signaling pathways (30). Nowadays, the most widely accepted hypotheses for AVMs formation in HHT1 and HHT2 involves a “first hit” (gene haploinsufficiency) based on the loss of one functional allele *Eng*^+/−^ (HHT1) or *ALK1*^+/−^ (HHT2), which causes a 50% reduction in protein expression, followed by a “second hit” based on an angiogenic trigger such as inflammation, hypoxia or vascular injury (31). Together, these events may induce a deficient endothelial function of ENG that could result in HHT vascular lesions. The relevant role of endoglin in the pathophysiology of the vascular system is illustrated by the phenotype of different HHT1 animal models (32). While endoglin heterozygous mice are viable and reproduce the vascular phenotype of HHT patients, total loss of ENG expression leads in mice to cardiovascular defects and embryonic death by mid-gestation (33–35). A conditional knock out mouse model for HHT1 has revealed that the arteriovenous malformations (AVMs), induced upon ENG loss, appear to be the result of delayed vascular remodeling and inappropriate ECs proliferation responses (36). In ENG null mice, vasculogenesis does not seem affected, suggesting that ENG is not required for initial differentiation of ECs or formation of a primitive vascular plexus. The yolk sac of *Eng*^−/−^ embryos has enlarged fragile vessels, while the embryo proper shows cardiac cushion defects and delayed maturation of major vessels (34). The reason why the total loss of ENG expression leads to cardiovascular defects and embryonic death is not completely understood. Some authors have postulated that the primary defect is in the heart (37), while others suggest that the loss of smooth muscle cells (SMCs) coverage of the endothelium could play a decisive role in the lethality of *Eng*^−/−^ animals (34).

## Endoglin Interacting Partners in the Vasculature

Previous studies reported that endothelial ENG is associated with several proteins critically involved in endothelial cell adhesion, proliferation, migration, angiogenesis, and vascular permeability, including integrins (38), zyxin (39), zyxin-related protein 1 (ZRP-1) (40), VEGF receptor type 2 (VEGFR-2) (41), beta-arrestin 2 (42), and vascular endothelial-cadherin (VE-cadherin) (43). It has been shown that VE-cadherin interacts with several components of the TGF-β receptor complex, including ENG, ALK1, and the TGF-β type II receptor TGFBR2 in adherents junctions. Of note, ENG, TGFBR2, and ALK1 also interact with p21-activated kinase (PAK-1), GTP-binding proteins, and Ras-related C3 botulinum toxin substrate 2 (Rac-2), involved in endothelial barrier maintenance, cytoskeletal remodeling and cell migration (44). Using *in vitro* ECs lines derived from ENG homozygous null, heterozygous, and control mouse embryos, Jerkic and Letarte have reported that ENG-deficient mouse embryonic ECs are hyper-permeable and unresponsive to stimulation by VEGF and TGF-β1, factors that regulate vascular permeability (45). Interestingly, VE-cadherin is barely detected in ENG-deficient ECs, a finding that could explain the EC junction destabilization and impairment of TGF-β signaling. Furthermore, permeability alterations in *Eng*^+/−^ mice are in line with findings in patients with Hereditary Hemorrhagic Telangiectasia type 1 (HHT1). Thus, ECs from HHT1 patients show a disorganized actin cytoskeleton prone to cell breaking with changes in shear stress and blood pressure (46). This reorganization of actin filaments in HHT1 might lead to vessel hemorrhages and eventual disappearance of the capillary network, as reported in this disorder. In line with the pathogenic hypothesis of an endothelial dysfunction in HHT1, the involvement of ENG in integrin-mediated trans-endothelial leukocyte trafficking was proposed (31). Accordingly, following inflammation triggering, the extracellular region of ENG binds to leukocyte integrin α5β1 and promotes leukocyte transmigration (31). In contrast, a different experimental approach showed an increased gut inflammation and myeloid infiltration in colitis *Eng*^+/−^ vs. control mice (47). Overall, this novel role of ENG may contribute to a better understanding of the inflammation process and the high incidence of infections in HHT patients (48). Because pericytes embrace the endothelium to regulate the blood retinal barrier, the role of endothelial endoglin in this context has also been analyzed. Thus, vascular permeability studies carried out *in vivo* showed that retinas of *Eng*^+/−^ heterozygous mice displayed higher retinal permeability than that of *Eng*^+/+^ mice, suggesting a destabilization of the endothelial barrier function due to ENG haploinsufficiency (23). The importance of the interaction between ECs and vascular mural cells is also underlined by studies showing that thalidomide stabilizes the vasculature in a mouse model of HHT1 (49). Indeed, pericyte recruitment and stabilization appears as a therapeutic strategy to reduce the severity of epistaxis in HHT (50). Thus, ENG, directly, or as a component of the TGF-β receptor complex may regulate ECs integrity, whereas its absence may result in vascular hyper-permeability, thus contributing to the fetal lethality associated with the homozygous null genotype (39, 40).

Although this review focuses on the role of endoglin in ECs, it is worth mentioning that endoglin is also expressed at lower levels in other cell types, some of which present in the vasculature as vascular SMCs, activated monocytes or mesenchymal cells (18, 51–53). However, a review on the endoglin role in these cell types deserves an independent study.

## Endoglin and Endothelial Colonies Forming Cells (ECFCs)

The formation of blood vessels from mesoderm is driven by the recruitment of undifferentiated cells which can differentiate into endothelial lineage. In the adult, populations of bone marrow-derived endothelial progenitor cells (EPCs) are mobilized into the circulation by stimuli such as estrogen and VEGF (54). Interestingly, human EPCs could derive from very small embryonic like stem cells (55) that can also give rise to hematopoietic cells (56). Two populations of EPCs have been differentially characterized from circulating endothelial progenitors: (i) early EPCs that exhibit a hematopoietic profile with a genomic fingerprint similar to monocytes; and (ii) ECFCs, also known as blood outgrowth endothelial cells (BOECs), which are considered late EPCs with an endothelial-like genomic profile (57). ECFCs display an intrinsic tube forming capacity *in vitro* and *in vivo* and they are involved in blood vessel formation and vascular repair (58, 59). For example, upon vascular injury, tissue perfusion of blood flow involves not only angiogenic sprouting of ECs from nearby intact vessels, but also the recruitment circulating ECFCs allowing the formation of new blood vessels (60). Acting at the interface between blood and tissues, ECFCs have a remarkable ability for migration and proliferation, being key cells in angiogenesis and vascular remodeling. It is therefore not surprising that perturbations in these critical ECFCs functions contribute to several vascular pathologies. Accordingly, ECFCs have opened a new area for treatment of cardiovascular diseases using cell therapy, advancing in the knowledge of vessel reconstruction ability in postnatal life. In this line, much effort has been recently devoted to identify novel molecular targets within ECFCs able to enhance their angiogenic potential. ECFCs are positive for endoglin (CD105), VE-cadherin (CD144), CD31, VEGFR2 (KDR), EGFL7, and CD146, and negative for CD45 and CD14 (58, 59). Among these, ENG is emerging as an interesting therapeutic target based on its involvement in angiogenesis and vascular remodeling (46, 61).

**Figure 2:**
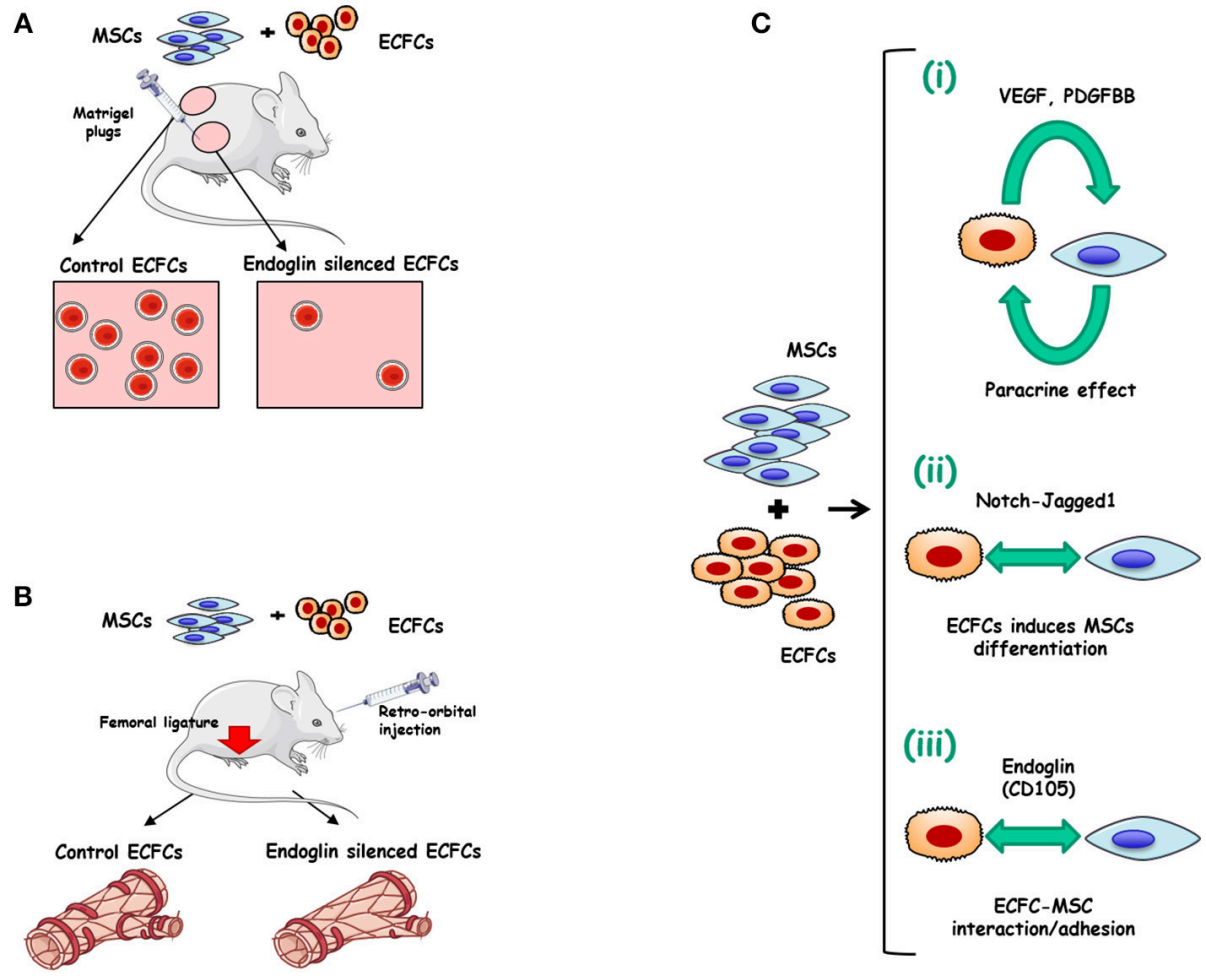
Endoglin in ECFCs. **(A)** Role of endoglin matrigel vascularization *in vivo*. Matrigel plugs were mixed with either ECFCs treated with control siRNA plus mesenchymal stem cells (MSCs) (left) or with ECFCs treated with endoglin specific siRNA plus MSCs (right). The Matrigel mixture was injected into nude mice and the number of vessels was analyzed after 1 week. Control plugs display a marked vascularization with functional vessels (presence of erythrocytes). Plugs with endoglin-silenced ECFCs display less vascularization than controls, suggesting an important role for this protein in cell adhesion (23). **(B)** MSCs combined with ECFCs, accelerate muscle recovery in a mouse model of hind limb ischemia, through an endoglin-dependent mechanism. After femoral ligature and retro-orbital injection of ECFC+MSC Doppler analysis shows a revascularization induced by co-injection of ECFCs plus MSCs (left). This synergistic effect is abolished when endoglin is previously silenced in ECFCs (right) (61). **(C)** Mechanisms involved in the synergy between ECFCs and MSCs. (i) Mutual paracrine effect between ECFCs and MSCs involving growth factors like VEGF and PDGFBB (76, 77). (ii) ECFCs-induced differentiation of MSCs into perivascular cells via Notch-Jagged1 (72) (iii) Adhesion between ECFCs and MSCs involving endoglin (61).

Many strategies of therapeutic revascularization, based on the administration of growth factors or stem/progenitor cells from diverse sources including EPCs, have been proposed and are currently being tested in patients with peripheral arterial disease or cardiac diseases (62). Some pretreatment of ECFCs with erythropoietin (63), fucoidan (64), soluble CD146 (65), tumor necrosis factor-α (TNF-α) (66), or platelet lysates (67, 68) have also been proposed to improve the therapeutic efficacy of *in vivo* administered ECFCs. Interestingly, integrins represent a major molecular determinant of EPCs function. More specifically, integrin α4β1 is a key regulator of EPCs retention and/or mobilization from the bone marrow, while integrins α5β1, α6β1, αvβ3, and αvβ5 are involved in EPCs homing, invasion, differentiation and paracrine factor production (69). The involvement of ECFCs in angiogenesis has been shown to be essential for ischemic disease recovery (70, 71). Furthermore, the differentiation of mesenchymal stem cells (MSCs) into mural cells appears to be stimulated by their co-culture with ECFCs, whereas overexpression of the Notch ligand Jagged1 in ECs further enhanced the differentiation of MSC into pericytes (72). This cooperation prompted some authors to postulate an improvement of ECFCs efficiency by co-injecting ECFCs and SMC progenitors (73). Therefore, a combined treatment of ECFCs with mesenchymal stem/progenitor cells (MSCs/MPCs) appears to operate synergistically, enhancing neovascularization compared to either individual cell population (74, 75). Recently, we proposed a role for ENG in the ECFCs-MSCs interplay involved in the revascularization process (61). Indeed, ENG silencing in ECFCs markedly inhibited ECFCs adhesion to MSCs *in vitro*, without affecting MSCs differentiation into perivascular cells or other mesenchymal lineages. Mesenchymal stem cells (MSCs) increase muscle recovery of ECFC in HLI model and we found that ENG-silenced ECFCs co-injected with MSCs in mice abolish beneficial effects of MSCs leading to decreased vessel density and foot perfusion upon ischemia. Our results suggest ENG involvement in the crosstalk between ECFCs and MSCs, leading to vessel formation and stabilization (51) (Figures 2A,B).

As postulated in Figure 2C, three steps may be involved during the formation of stable vessels by ECFC and MSC and/or perivascular cells: (i) Mutual paracrine effects by VEGF and/or PDGF-BB (76, 77). (ii) ECFCs-driven perivascular maturation of MSC and/or SMC progenitors via the notch-dependent pathway; and (iii) Adhesion between ECFCs and perivascular cells in an ENG-dependent manner. The relevance of endothelial ENG adhesive properties in mature vessel formation in mice (61) is in agreement with a previous report on the active role of human ENG in the adhesion between mature ECs and vascular mural cells (23).

Relevance of ENG in ECFC has been demonstrated also in ECFCs isolated directly from HHT1 patients. Indeed, these ECFCs have disorganized and depolymerized actin fibers and impaired tube formation, as compared to healthy ECFCs (46). The molecular basis for the abnormal behavior of HHT1 ECFCs appears to be mediated, at least in part, by the regulatory role of endoglin and we can speculate *via* its interaction with zyxin family members involved in the actin cytoskeletal organization as it was proposed for endothelial cells (39, 40). Thus, the ENG cytoplasmic domain binds to zyxin and ZRP1, which concentrate at focal adhesions within actin polymerization points. Consequently, the decrease of ENG levels in HHT1 ECFCs could be related to the cytoskeleton alteration.

## Conclusion

Blood vessel formation and remodeling are essential processes in the maintenance of tissue homeostasis and function, and therefore, their alteration causes a variety of pathologic conditions. ENG involvement in the function of mature and progenitor endothelial cells is a hot topic not completely understood and developed yet. Recent findings underline the importance of ENG not only as a co-receptor for several ligands of the TGF-β family, but also as a molecule involved in cell adhesion, which can regulate ECFCs behavior in the context of angiogenic processes. It is recognized that in mature ECs, ENG plays a key role in angiogenesis and blood vessel homeostasis, becoming a potential therapeutic target for pro- and anti-angiogenic approaches in the treatment of diseases such as HHT, cancer, preeclampsia, diabetes complications or post-ischemic disease. While ENG expression is relatively low in quiescent ECs, it is upregulated in the active endothelium involved in angiogenesis and vessel remodeling and permeability. In these processes, endothelial ENG regulates ECs proliferation, migration, and actin cytoskeletal organization, leukocyte extravasation, and interaction with mural cells. In addition, a role for ENG in platelet-endothelium interaction, including the resistance of adherent platelets to shear under inflammatory conditions, was also recently proposed, opening new perspectives on ENG functions.

This review highlights the emerging roles of endothelial ENG as a cell adhesion molecule in mature and progenitor endothelial cells interacting with vascular mural cells, leukocytes, and platelets, independently or in parallel to its TGF-β co-receptor role.

While evidence-based therapeutics are coming into play in the traditionally empirical base for endoglin related protein derivatives, future studies of ENG in endothelial progenitor cells may pave the way for a better understanding of its function in the vascular system and for the development and understanding of the use of these immature cells as a cell therapy product.

## Author Contributions

ER wrote the manuscript. CB provided helpful suggestions and contributed to writing the manuscript. DS wrote the manuscript and provided funding support.

### Conflict of Interest Statement

The authors declare that the research was conducted in the absence of any commercial or financial relationships that could be construed as a potential conflict of interest.
